# A Transcriptomic Study of Maternal Thyroid Adaptation to Pregnancy in Rats

**DOI:** 10.3390/ijms161126030

**Published:** 2015-11-13

**Authors:** Ji-Long Liu, Tong-Song Wang, Miao Zhao, Ying Peng, Yong-Sheng Fu

**Affiliations:** 1 College of Veterinary Medicine, South China Agricultural University, Guangzhou 510642, China; zm1586186423@126.com (M.Z.); yingpeng2013@126.com (Y.P.); yshf2013@126.com (Y.-S.F.); 2 Department of Biology, Shantou University, Shantou 515063, China; iamwangtongsong@163.com

**Keywords:** thyroid, pregnancy, adaptation, RNA-seq

## Abstract

Thyroid disorders are relatively frequently observed in pregnant women. However, the impact of pregnancy on maternal thyroid has not been systematically evaluated. In the present study, using the rat as an animal model, we observed that the weight of maternal thyroid increased by about 18% in late pregnancy. To gain an insight into the molecular mechanisms, we took advantage of RNA-seq approaches to investigate global gene expression changes in the maternal thyroid. We identified a total of 615 differentially expressed genes, most of which (558 genes or 90.7%) were up-regulated in late pregnancy compared to the non-pregnant control. Gene ontology analysis showed that genes involved in cell cycle and metabolism were significantly enriched among up-regulated genes. Unexpectedly, pathway analysis revealed that expression levels for key components of the thyroid hormone synthesis pathway were not significantly altered. In addition, by examining of the promoter regions of up-regulated genes, we identified MAZ (MYC-associated zinc finger protein) and TFCP2 (transcription factor CP2) as two causal transcription factors. Our study contributes to an increase in the knowledge on the maternal thyroid adaptation to pregnancy.

## 1. Introduction

A wide range of maternal physiological adaptations occurs during pregnancy. Well-documented examples include liver weight gain [1], pancreatic β cell mass expansion [2], cardiac hypertrophy [3] and immune system recalibration [4]. Although these physiological changes are crucial for the establishment and maintenance of pregnancy, they may also lead to maternal pathogenesis by acting as a stressor.

In particular, the maternal thyroid is substantially challenged during pregnancy. Pregnancy-induced thyroid enlargement has been recognized for thousands of years [5]. Studies have confirmed that thyroid enlargement is evident in areas of moderate or low iodine but not in those with sufficient iodine [6]. The thyroid hormone economy is altered during pregnancy. Serum total triiodothyronine (T_3_) and thyroxine (T_4_) levels increase by 50% during pregnancy as a result of elevated levels of thyroid hormone carrier proteins [7]. The production of T_3_ and T_4_ is stimulated by thyroid-stimulating hormone (TSH) from pituitary. The levels of serum TSH decline in the first trimester and elevate in the second and third trimesters [7]. The decrease of serum TSH in the first trimester is explained by the high level of serum human chorionic gonadotropin (hCG), a glycoprotein hormone that has structural similarity to TSH and is cross-reactive to the TSH receptor [8,9,10]. Notably, thyroid disorders are relatively frequently observed in pregnant women: hypothyroidism is present in 2%–3% of pregnant women [11,12], overt hyperthyroidism occurs in approximately 0.5% of pregnant women [13], and the prevalence of postpartum thyroiditis is approximately 5.4% [14]. These pregnancy-dependent thyroid disorders may jeopardize maternal and fetal health.

However, the impact of pregnancy on maternal thyroid has not been systematically evaluated. In the present study, using the rat as an animal model, we observed that the weight of maternal thyroid increased by about 18% in late pregnancy. To gain an insight into the molecular mechanisms, we took advantage of the RNA-seq approach to investigate the global gene expression changes in the maternal thyroid. RNA-seq is a highly accurate tool for quantifying global gene expression levels. In contrast to microarray, the main advantages of RNA-seq are that it can detect un-annotated transcripts [15], discriminate very similar sequences [16], and exceed the quantification dynamic range of microarray [17]. Our study provides insights into the maternal thyroid adaptation to pregnancy.

## 2. Results

### 2.1. Maternal Thyroid Weight Changes during Pregnancy in Rats

**Figure 1:**
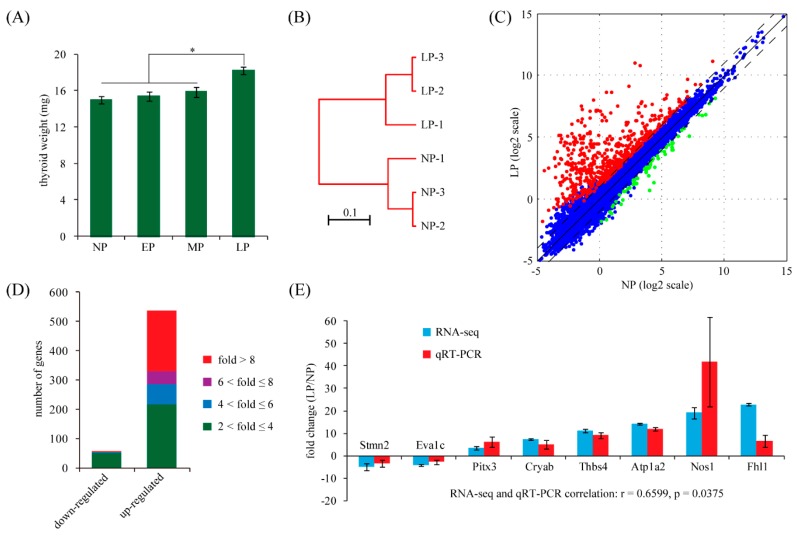
Identification of differentially expressed genes in maternal thyroid in late pregnancy. (**A**) Maternal thyroid weight responses to pregnancy in rats. Thyroid tissues were collected from non-pregnant (NP) and pregnant rats on Days 8 (early pregnancy, EP), 12 (middle pregnancy, MP) and 18 (late pregnancy, LP) of pregnancy. Absolute thyroid weight is used. Each bar represents at least three animals. * *p* < 0.05; (**B**) Cluster dendrogram of RNA-seq data generated from six samples. The Pearson correlation distance measure and the average linkage clustering algorithm were used. The plot was generated by using bioinformatics toolbox 3.3 of MATLAB (MathWorks); (**C**) Scatter plot depicting the expression profiles of all genes. FPKM values from RNA-Seq are plotted in log2 scale. Dash lines indicate the two-fold difference boundaries. Non-changed genes were shown in blue color, while differently expressed genes (fold change > 2 and adjusted *p* < 0.05) were denoted in red or green; (**D**) Distribution of fold change values among down-regulated and up-regulated genes; (**E**) Validation of selected genes identified by RNA-Seq using qRT-PCR. Fold-change values determined by both RNA-seq and qRT-PCR were presented as the mean ± SD. Statistical significance was reached at *p* < 0.05 for all genes. Stmn2, stathmin 2; Eva1c, eva-1 homolog C; Pitx3, paired-like homeodomain 3; Cryab, crystallin αB; Thbs4, thrombospondin 4; Atp1a2, ATPase Na^+^/K^+^ transporting α2 polypeptide; Nos1, nitric oxide synthase 1 neuronal; Fhl1, four and a half LIM domains 1.

To determine whether there is an adaptive response of the maternal thyroid to pregnancy, we first evaluated the changes in maternal thyroid weight at different stages of pregnancy in rats. Absolute thyroid weight was used to avoid errors in relative thyroid weight due to varying fetus mass and maternal weight gain during pregnancy. Maternal thyroids were collected from non-pregnant (NP) and pregnant rats (early pregnancy: EP, Day 8; middle pregnancy: MP, Day 12; late pregnancy: LP, Day 18) and weighed. We observed that the weight of maternal thyroid from EP and MP was not significantly different from NP rats. However, the weight of maternal thyroid then increased by about 18% in LP rats (Figure 1A), indicating an evident response of the maternal thyroid in late pregnancy.

### 2.2. Identification of Differentially Expressed Genes in Maternal Thyroid in Late Pregnancy

To identify potential regulators of the maternal thyroid, gene expression profiles of non-pregnant (NP) and late pregnant (LP) rats were analyzed using the RNA-seq approach. The RNA-seq raw data were deposited in Gene Expression Omnibus database (GSE73307). We obtained a total of 25.4 million reads, 83.1% of which were uniquely mapped to the rat genome. Mapped reads were used to estimate normalized transcription level as fragments per kilobase of transcript per million mapped fragments (FPKM). It has been estimated that a gene with FPKM value of 1 is approximately equivalent to one copy per cell [16]. Whole-transcriptome clustering analysis demonstrated that the NP samples were readily separated from the LP ones (Figure 1B). This result confirmed that maternal thyroid gene expression is systematically altered in LP.

The DESeq package was used to test for differential expression of genes between NP and LP. Using a fold change cutoff of two and an adjusted *p*-value cutoff of 0.05, we identified a total of 615 differentially expressed genes (Figure 1C). Among them, 57 genes were down-regulated and 558 genes were up-regulated in LP in comparison to NP (Table S1). The range of fold changes was 2.1–10.0 and 2.0–491.5 for down-regulated and up-regulated genes, respectively (Figure 1D).

We randomly selected eight genes with various fold changes and validated them by quantitative RT-PCR (qRT-PCR) in an independent set of biological replicates. The qRT-PCR results for all selected genes were concordant with the trend estimated by RNA-Seq. Moreover, the statistical significance was reached at *p* < 0.05 for all tested genes (Figure 1E). In general, the qRT-PCR and RNA-Seq results showed a statistically significant correlation (Pearson correlation, *r* = 0.6599, *p* = 0.0375). This result suggested that our high throughput RNA-seq data are as reliable as conventional qRT-PCR.

### 2.3. Gene Ontology (GO) Analysis of Differentially Expressed Genes in Maternal Thyroid in Late Pregnancy

**Figure 2:**
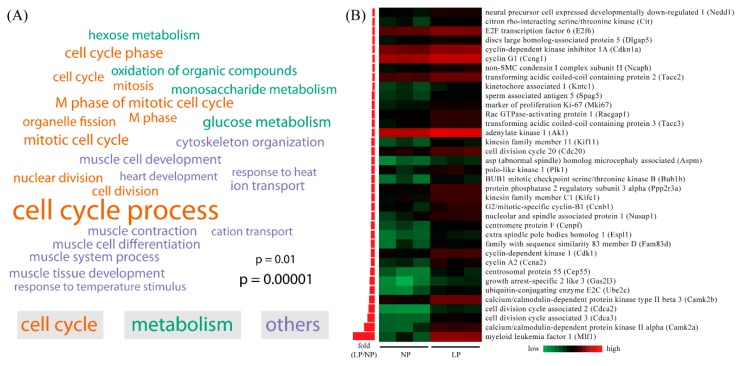
Gene ontology (GO) analysis of differentially expressed genes. (**A**) Word cloud of enriched GO terms in the biological process category for differentially expressed genes. The enrichment test was performed using the DAVID tool and the significance cutoff for adjusted *p*-value was set at 0.01. The font sizes in the word cloud are proportional to –log10 of adjusted *p*-value for each enriched GO terms; (**B**) Heatmap of the 36 differentially expressed genes that fall into the biological process of cell cycle. FPKM values were log-transformed and centered relative to the median. The fold change values are presented as bars on the right of the heatmap in ascending order. The range of fold changes is 2.00–74.66.

Gene ontology (GO) and pathway analysis was performed by using the DAVID (Database for Annotation, Visualization and Integrated Discovery) tool. Because down-regulated genes took up a small portion of the differentially expressed genes, only up-regulated genes were subject to GO and pathway analysis. Enriched GO terms are classified according to biological process annotations (Figure 2A). A total of 25 terms were significantly enriched, including cell cycle process (*p* = 6.36 × 10^−6^), mitotic cell cycle (*p* = 1.07 × 10^−5^), cell cycle phase (*p* = 2.15 × 10^−5^), M phase of mitotic cell cycle (*p* = 2.69 × 10^−5^), M phase (*p* = 7.89 × 10^−5^), organelle fission (*p* = 8.46 × 10^−5^), cell cycle (*p* = 1.31 × 10^−4^), nuclear division (*p* = 1.25 × 10^−4^), mitosis (*p* = 1.25 × 10^−4^), cell division (*p* = 3.39 × 10^−4^), glucose metabolic process (*p* = 5.23 × 10^−5^), hexose metabolic process (*p* = 9.99 × 10^−5^), oxidation of organic compounds (*p* = 1.27 × 10^−4^), monosaccharide metabolic process (*p* = 3.62 × 10^−4^), cytoskeleton organization (*p* = 8.91 × 10^−5^), muscle tissue development (*p* = 9.43 × 10^−5^), ion transport (*p* = 1.49 × 10^−4^), muscle cell differentiation (*p* = 1.92 × 10^−4^), muscle system process (*p* = 3.61 × 10^−4^), muscle contraction (*p* = 7.49 × 10^−4^), muscle cell development (*p* = 8.36 × 10^−4^), response to heat (*p* = 2.26 × 10^−3^), response to temperature stimulus (*p* = 7.49 × 10^−3^), heart development (*p* = 7.57 × 10^−3^) and cation transport (*p* = 9.56 × 10^−3^). Among all these GO terms, 10 terms were associated with cell cycle. We next extracted 36 genes associated with cell cycle out of the 615 differentially expressed genes. All of the 36 genes were up-regulated (Figure 2B). This result indicates an increase of proliferation in the maternal thyroid in LP.

**Figure 3:**
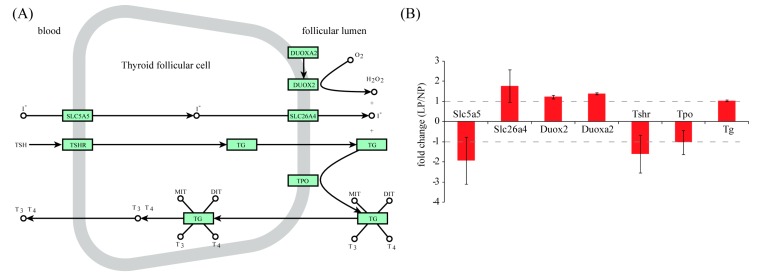
Gene expression analysis of thyroid hormone synthesis pathway. (**A**) Visualization of the thyroid hormone synthesis pathway. Rectangular nodes represent genes and circular nodes denote small molecules. Edges represent reactions derived from KEGG pathway rno04918. This graph is generated using the Cytoscape software. TSH, thyroid stimulating hormone; TSHR, thyroid stimulating hormone receptor. SLC5A5, solute carrier family 5 member 5; SLC26A4, solute carrier family 26 member 4; TG, thyroglobulin; TPO, thyroperoxidase; DUOXA2, dual oxidases A2; DUOX2, dual oxidases 2; (**B**) Bar plot showing fold change values based on RNA-seq data. None of these genes were differentially expressed according to the criteria of fold change > 2 and adjusted *p*-value < 0.01.

Pathway analysis was performed using the KEGG (Kyoto Encyclopedia of Genes and Genomes) pathway database. The calcium signaling pathway was the only pathway that was significantly enriched among up-regulated genes (*p* = 1.34 × 10^−3^). We are particularly interested in the thyroid hormone synthesis pathway in follicular cells of the thyroid. Iodine is an important element for the synthesis of thyroid hormones. In order to maintain high concentrations of Iodine, iodide (I^−^) is continuously transported into follicular cells from serum by the sodium/iodide symporter (solute carrier family 5 member 5, SLC5A5). Pendrin (solute carrier family 26 member 4, SLC26A4), which is expressed at the apical membrane of follicular cells, transports I^−^ from the cytoplasm into the follicle lumen. Thyroid hormones are synthesized on surface of a tyrosine-enriched protein, thyroglobulin (TG), which is produced in follicular cells and subsequently secreted into the follicular lumen. The key enzyme for thyroid hormone synthesis is thyroperoxidase (TPO). Under the oxidative environment created by the dual oxidases (DUOXA2 and DUOX2), TPO is able to oxidize I^−^ into I_2_ and link the resulting I_2_ with the tyrosine residues on TG to generate monoiodotyrosine (MIT) and diiodotyrosine (DIT). TPO is also responsible for combining MIT and DIT to generate T_3_ or T_4_ on the surface of the TG protein. Finally, stimulated by thyroid stimulating hormone (TSH), T_3_/T_4_-loaded TG is endocytosed by follicular cells and subsequently broken down by the lysosome. T_3_ and T_4_ are then released into the circulation. Key components of the thyroid hormone synthesis pathway are illustrated in Figure 3A. Based on RNA-seq data, all these genes did not exceed a two-fold change (Figure 3B), suggesting that thyroid hormone synthesis is not altered in LP.

### 2.4. Identification of Transcription Factor Binding Sites (TFBS) in the Promoters of Up-Regulated Genes

**Figure 4:**
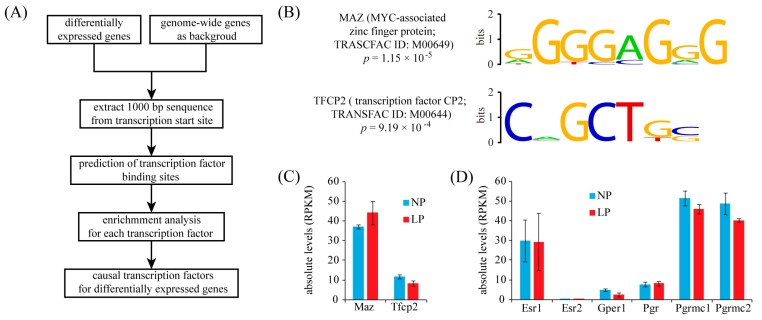
Enrichment of transcription factor binding sites (TFBS) in up-regulated genes. (**A**) The workflow for TFBS enrichment analysis; (**B**) The sequence logo display for two transcription factors whose binding sites are significantly enriched in up-regulated genes; (**C**) Bar plot showing absolute expression levels for MAZ and TFCP2; (**D**) Bar plot showing absolute expression levels for estrogen receptors and progesterone receptors. Esr1, estrogen receptor α; Esr2, estrogen receptor β; Gpre1, G protein-coupled estrogen receptor 1; Pgr, progesterone receptor; Pgrmc1, progesterone receptor membrane component 1; Pgrmc2, progesterone receptor membrane component 2.

We hypothesized that the up-regulated genes may also share common regulatory mechanisms such as transcription factor binding sites (TFBS) in their promoter regions. We focused on the proximal promoter sequences (1 kb upstream of transcription start sites). Potential TFBS sites were detected using the TESS software configured with position-weigh matrices (PWMs) from the TRANSFAC database. Only hits with a relative score above 0.9 were kept. Then statistically enriched transcription factors were determined using a hypergeometric test with Benjamini-Hochberg multiple test correction. The computational pipeline for TFBS analysis is depicted in Figure 4A. In the end, only two transcription factors, Myc-associated zinc finger protein (MAZ) and transcription factor CP2 (TFCP2), were significantly enriched (*p* < 0.05) (Figure 4B). Based on RNA-seq data, both MAZ and TFCP2 were expressed at median levels and no difference was detected between LP and NP (Figure 4C).

Pregnancy is accompanied by a dramatic increase of estrogen and progesterone levels in the serum. Estrogen and progesterone exert their actions either by activating nuclear receptors (ESR1/ESR2 and PGR) [18,19] or by interacting with membrane receptors (GPER1 and PGRMC1/PGRMC2) [20,21]. We checked if these hormone receptors were expressed in the thyroid. Using 1 FPKM as the cutoff value, all these receptors were present in the thyroid, except ESR2 (Figure 4D). Because nuclear receptors ESR1 and PGR were not enriched in the TFBS analysis, we supposed that maternal hormones may up-regulate gene expression in the thyroid mainly via membrane receptors GPER1, PGRMC1 and PGRMC2.

## 3. Discussion

In the present study, we utilized the rat as an animal model to study maternal thyroid adaptation to pregnancy. We found that the weight of maternal thyroid from pregnant rats was not significantly different from that from non-pregnant rats until late pregnancy. Thus, RNA-seq was employed to characterize the maternal thyroid transcriptome in late pregnancy. We identified a total of 615 differentially expressed genes, most of which (558 genes or 90.7%) were up-regulated in late pregnancy compared to the non-pregnant control. Among these differentially expressed genes, we validated eight genes using qRT-PCR. The expression trend of these genes measured by qRT-PCR was consistent with RNA-seq, suggesting that our data were of high quality. Our study provides a valuable resource for the in-depth understanding of the maternal thyroid adaptation to pregnancy.

Based on gene ontology analysis, a total of 25 terms were significantly enriched among up-regulated genes, indicating that maternal thyroid adaptation to pregnancy invokes many genes with a wide range of different functions. Among all these GO terms, 10 terms were associated with cell cycle and four terms were associated with metabolism. Of special interest, the top 10 up-regulated genes were: Ckmt2 (creatine kinase mitochondrial 2), Actn2 (actinin α2), Tcap (titin-cap), Cacna1s (calcium channel voltage-dependent L type α1S subunit), Xirp2 (xin actin-binding repeat containing 2), Hfe2 (hemochromatosis type 2), Atp2a1 (ATPase Ca^2+^ transporting cardiac muscle fast twitch 1), Cox6a2 (cytochrome c oxidase subunit VIa polypeptide 2), Trim54 (tripartite motif-containing 54) and Nrap (nebulin-related anchoring protein). Ckmt2 is the most highly up-regulated gene (fold change = 491.5). Ckmt2 is involved in the metabolism process, responsible for the transfer of high energy phosphate from the mitochondrion to creatine in the cytosol. It has been shown that Ckmt2 is up-regulated in Graves’ thyroid tissues [22]. Thus, elevated Ckmt2 expression may contribute largely to pregnancy-induced proliferation of the maternal thyroid. Another gene involved in the metabolism process is Cox6a2, whose function in thyroid has not been defined yet. Actn2 is the second most highly up-regulated gene. Actn2 is associated with cytoskeleton and it is co-localized with β-catenin at the cell junction in thyroid follicular cells [23]. A gene profiling study has shown that Actn2 is over-expressed in oncocytic and papillary thyroid carcinoma [24]. Beyond Actn2, four other top-ranked genes (Tcap, Xirp2, Trim54 and Nrap) are also associated with cytoskeleton. The remaining three genes (Hfe2, Cacna1s and Atp2a1) appear to play a role in signaling transduction. Notably, although a relatively large number of cell cycle genes were found in up-regulated genes, they were not among the top-ranked genes. The highest rank is 51 for Mlf1 (myeloid leukemia factor 1) with a fold change value of 74.6. We also performed GO analysis on down-regulated genes; however, due to the small number of genes, no enriched GO term was detected. The top 10 down-regulated genes were: Slc2a10 (solute carrier family 2 member 10 or facilitated glucose transporter), Fmo2 (flavin containing monooxygenase 2), Stmn2 (stathmin 2), Cacng5 (calcium channel voltage-dependent gamma subunit 5), Fmo3 (flavin containing monooxygenase 3), Snx22 (sorting nexin 22), Eva1c (eva-1 homolog C), Bhlhe22 (basic helix-loop-helix family member e22), Ccdc152 (coiled-coil domain containing 152) and Cyp26b1 (cytochrome P450 family 26 subfamily b polypeptide 1). Among these genes, Slc2a10, Fmo2, Fmo3 and Cyp26b1 are involved in the metabolism process. Stmn2 is part of cytoskeleton. The other top-ranked down-regulated genes, Cacng5, Snx22, Eva1c, Bhlhe22 and Ccdc152, may participate in signaling transduction.

In pathway analysis, we focused on the thyroid hormone synthesis pathway. Unlike the case of humans, the accumulation of iodine and the concentration of total T_3_ and T_4_ in serum are both reduced during late pregnancy in rats [25]. Meanwhile, the serum TSH levels are slightly higher on 18–20 days of pregnancy than in non-pregnant rats [26]. Taken together, it appears that the increase of TSH is due to the effect of negative feedback in the hypothalamic-pituitary-thyroid axis. Unexpectedly, pathway analysis revealed that expression levels for key components of the thyroid hormone synthesis pathway were not significantly altered. However, proteins but not mRNAs are functional in cells. Due to post-transcriptional regulations, the correlation between mRNA and protein expression levels is usually modest [27]. Therefore, further validation at the protein expression level may be needed.

In the present study, two transcription factors, MAZ and TFCP2, were identified as shared transcription factors for up-regulated genes. Thus, it is likely that these two transcription factors may orchestrate a large part of maternal thyroid adaptation to pregnancy. According to RNA-seq data, both MAZ and TFCP2 were expressed at median levels in the thyroid. MAZ binds the promoter of MYC (v-myc avian myelocytomatosis viral oncogene homolog) to regulate transcriptional activity [28,29]. MYC, a transcription factor from the basic helix-loop-helix leucine zipper (bHLHZ) family, is able to form heterodimers with other family members, such as MAX (MYC associated factor X). The MAX/MYC dimer promotes cell proliferation and apoptosis [30]. A study has demonstrated that MAZ is able to up-regulate TSG101 (tumor susceptibility 101) gene expression in human thyroid by binding to its proximal promoter [31]. TFCP2 is also called late SV40 factor (LSF). TFCP2 binds a variety of promoters for cellular and viral genes, including fibrinogen, α-globin, SV40 and HIV-1 [32]. Currently, little is known about the role of TFCP2 in regulating thyroid gene expression. Interestingly, we found that the gene ontology term cell cycle is significantly enriched in MAZ target genes, whereas both cell cycle and metabolism are significantly enriched in TFCP2 target genes, suggesting that MAZ and TFCP2 may be key regulators of thyroid adaptation to pregnancy (shown in Table S2). Hence, these two transcription factors deserve further investigation. Pregnancy is characterized by a dramatic increase in maternal hormones, including estrogen and progesterone. According to RNA-seq data, nuclear receptors, ESR1 and PGR, were expressed in the thyroid. However, they were not enriched in the transcription factor binding site (TFBS) analysis. Additionally, we found that membrane receptors GPER1, PGRMC1 and PGRMC2 were also expressed in the thyroid. Thus, it is tempting to speculate that maternal estrogen and progesterone may up-regulate gene expression in the thyroid mainly via membrane receptors.

In conclusion, in the present study, using RNA-seq, we identified differentially expressed genes in the maternal thyroid in rats during late pregnancy. Our study contributes to an increase in the knowledge on the maternal thyroid adaptation to pregnancy. Deciphering the drivers of these differentially expressed genes may open the opportunity to devise therapeutic methods to prevent and treat pregnancy-dependent thyroid disorders.

## 4. Materials and Methods

### 4.1. Sample Collection

Adult pregnant Sprague-Dawley rats and their non-pregnant controls were utilized in this study. Pregnant rats were obtained by co-caging two females with a male overnight. The presence of a vaginal plug was checked the next morning as an indicator of successful mating. Thyroid tissues were obtained from non-pregnant (*n* = 3) rats and pregnant rats on Days 8 (*n* = 3), 12 (*n* = 4) and 18 (*n* = 3) of pregnancy (day 1 = day of vaginal plug). All collected tissues were then snap-frozen in liquid nitrogen and stored at −80 °C until use. All animal procedures were approved by the Institutional Animal Care and Use Committee of South China Agricultural University.

### 4.2. RNA-Seq Analysis

Total RNA from thyroid samples was extracted using the TRIzol reagent (invitrogen, Carlsbad, CA, USA). RNA purity and integrity were assessed by using the ND-1000 Nanodrop and the Agilent 2200 TapeStation, respectively. The following parameters were set for RNA quality control: A260/A280 ratio ≥ 1.8, A260/A230 ratio ≥ 2.0 and RIN value ≥ 7.0. The TruSeq RNA sample preparation kit (Illumina, San Diego, CA, USA) was used for RNA-seq library preparation. Finally, sequencing of the libraries was conducted on an Illumina HiSeq™ 2500 system.

A computational pipeline was used to process RNA-seq data. Sequence data were mapped to rat reference genome rn5 with Tophat v1.4.0 [33] with default parameters. HTSeq-count [34] was subsequently employed to convert aligned short reads into read counts for each gene model in the ENSEMBL database release 81 [35]. Differential expression between was assessed by DEseq [36] using read counts as input. The Benjamini–Hochberg multiple test correction method was enabled. Differentially expressed genes were chosen according to the criteria of fold change > 2 and adjusted *p*-value < 0.05.

### 4.3. Validation by Quantitative RT-PCR

Total RNA was extracted by using the TRIzol reagent (Invitrogen). Potential genomic DNA contamination was eliminated by DNase I (Invitrogen) treatment. Reverse transcription was performed using the PrimeScript reverse transcriptase reagent kit (TaKaRa, Dalian, China). Real-time PCR was carried out using the SYBR Premix Ex TaqTM kit (TaKaRa) on the Rotor-Gene 3000A system (Corbett Research, Sydney, Australia). Using Rpl7 as the reference gene, relative expression levels of the selected genes were calculated using the 2^−ΔΔ*C*t^ method. Primer sequences used in this study were listed in Table S3.

### 4.4. Functional Clustering Analysis

Gene ontology (GO) and pathway analysis was performed by using the DAVID tool [37]. The significance cutoff for Benjamini–Hochberg adjusted *p*-value was set at 0.01. The R package wordcloud was used to generate word cloud for significantly enriched GO terms. The font sizes in the word cloud were proportional to −log10 of adjusted *p*-value for each enriched GO terms.

### 4.5. Analysis of Transcription Factor Binding Sites (TFBS)

Promoter sequences (1 kb upstream of the transcription start site) were extracted for each gene. The TESS software version 6.0 [38] was used to scan these promoter sequences for matches of position-weigh matrices (PWMs) from the TRANSFAC database [39]. Only hits with a relative score above 0.9 were kept. We calculated the percentage of up-regulated genes with binding site for each transcription factor. All genes in the genome served as the background. To test for enrichment, a hypergeometric test with Benjamini–Hochberg multiple test correction was conducted using in-house PERL scripts. In the end, *p* < 0.05 was used as significance threshold to identify enriched transcription factors.

## Supplementary Materials

Supplementary materials can be found at http://www.mdpi.com/1422-0067/16/11/26030/s1.
